# Metabolic and transcriptional elucidation of the carotenoid biosynthesis pathway in peel and flesh tissue of loquat fruit during on-tree development

**DOI:** 10.1186/s12870-017-1041-3

**Published:** 2017-06-14

**Authors:** Margarita Hadjipieri, Egli C. Georgiadou, Alicia Marin, Huertas M. Diaz-Mula, Vlasios Goulas, Vasileios Fotopoulos, Francisco A. Tomás-Barberán, George A. Manganaris

**Affiliations:** 1 Department of Agricultural Sciences, Biotechnology and Food Science, Cyprus University of Technology, 3603 Lemesos, Cyprus; 2 Quality, Safety, and Bioactivity of Plant Foods, CEBAS-CSIC, P.O. Box 164, Espinardo, Murcia, Spain

**Keywords:** *Eriobotrya japonica*, Developmental stages, Maturation, Ripening, β-carotene, β-cryptoxanthin, Lutein, LC-MS, Biosynthetic pathway

## Abstract

**Background:**

Carotenoids are the main colouring substances found in orange-fleshed loquat fruits. The aim of this study was to unravel the carotenoid biosynthetic pathway of loquat fruit (cv. ‘Obusa’) in peel and flesh tissue during distinct on-tree developmental stages through a targeted analytical and molecular approach.

**Results:**

Substantial changes regarding colour parameters, both between peel and flesh and among the different developmental stages, were monitored, concomitant with a significant increment in carotenoid content. Key genes and individual compounds that are implicated in the carotenoid biosynthetic pathway were further dissected with the employment of molecular (RT-qPCR) and advanced analytical techniques (LC-MS). Results revealed significant differences in carotenoid composition between peel and flesh. Thirty-two carotenoids were found in the peel, while only eighteen carotenoids were identified in the flesh. *Trans*-lutein and *trans*-β-carotene were the major carotenoids in the peel; the content of the former decreased with the progress of ripening, while the latter registered a 7.2-fold increase. However, carotenoid profiling of loquat flesh indicated *trans*-β-cryptoxanthin, followed by *trans*-β-carotene and 5,8-epoxy-β-carotene to be the most predominant carotenoids. High amounts of *trans*-β-carotene in both tissues were supported by significant induction in a chromoplast-specific *lycopene β-cyclase (CYCB)* transcript levels. *PSY1*, *ZDS*, *CYCB* and *BCH* were up-regulated and *CRTISO*, *LCYE*, *ECH* and *VDE* were down-regulated in most of the developmental stages compared with the immature stage in both peel and flesh tissue. Overall, differential regulation of expression levels with the progress of on-tree fruit development was more evident in the middle and downstream genes of carotenoid biosynthetic pathway.

**Conclusions:**

Carotenoid composition is greatly affected during on-tree loquat development with striking differences between peel and flesh tissue. A link between gene up- or down-regulation during the developmental stages of the loquat fruit, and how their expression affects carotenoid content per tissue (peel or flesh) was established.

**Electronic supplementary material:**

The online version of this article (doi:10.1186/s12870-017-1041-3) contains supplementary material, which is available to authorized users.

## Background

Loquat (*Eriobotrya japonica* Lindl) is a member of the Rosaceae family that is commercially cultivated in many countries [1–3], being highly appreciated for its light, refreshing taste [2, 4]. Therefore, although initially considered as an underutilized crop, nowadays loquat can gain added value as it is available during late winter- early spring period [5]. The loquat tree has three flushes of growth per year, and the principal tree growth can be separated into 8 distinct developmental stages [2]. In particular, under Mediterranean weather conditions, the tree blooms between October and early November and its fruit develops through winter, ripening from early February until May. Fruit is usually consumed fresh, but it is also known to be used processed into jam, jelly, wine, syrup, and juice. It is also known that the leaves, flowers and fruits are traditionally used in Chinese medicine since they are linked with health-promoting properties [1, 6].

Carotenoids play an important role in loquat, as they affect organoleptic characteristics and health properties of the fruit. In particular, carotenoids are the main pigments in loquat and impact flavor acceptability, since they are precursors of important volatile flavor compounds [7]. Regarding nutraceutical properties of fruit carotenoids, a significant number of studies depicted their beneficial effect to the promotion of health, including the prevention and/or treatment of chronic and cardiovascular diseases [8]. In particular, fruits rich in carotenoids are directly connected to the prevention of inflammation and cataract [1, 9, 10] and are also known to enhance immune responses [1, 9, 11]. Carotenoid profile in loquat is influenced by maturity stage, environmental and most promptly genetic factors. Loquat cultivars have been segregated to white- and red-fleshed [10, 12]. However, segregation of loquat cultivars based on their flesh color can be confusing, since additionally the terms yellow- and orange-fleshed are being used. White-fleshed cultivars have a creamy, pale yellow color, while the terms red- and orange-fleshed can be considered as synonymous. The latter type cultivars have higher carotenoid concentrations than the lighter coloured ones [1, 5, 9, 10, 12].

**Fig. 1:**
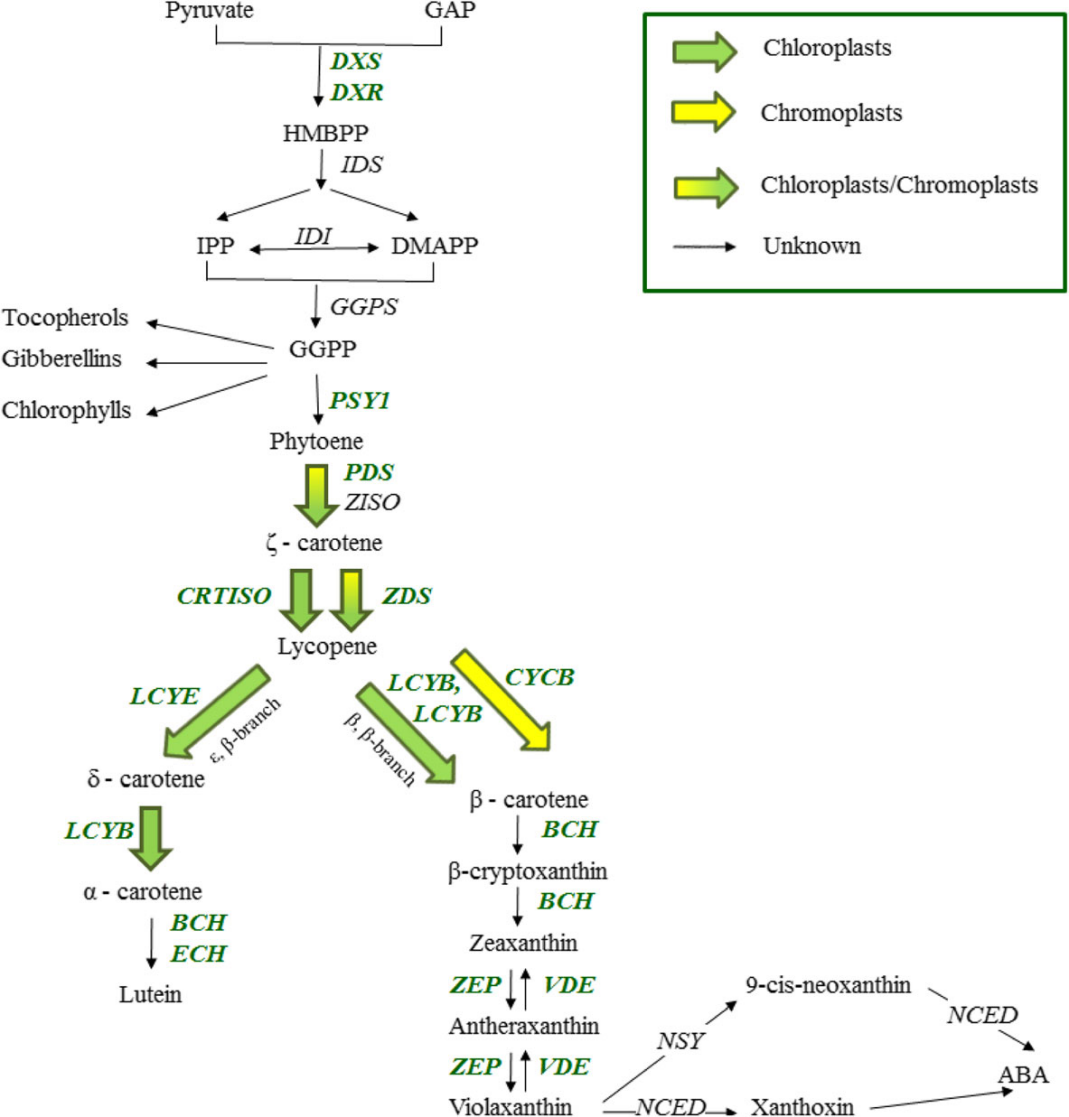
Carotenoid biosynthetic pathway in loquat fruit. Genes examined are in *green italic bold letters*. The enzymes/genes are: DXS, 1-deoxy-D-xylulose 5-phosphate-synthase; DXR, DXP reductoisomerase; IDS, isopentenyl pyrophosphate synthase; IDI, isopentenyl pyrophosphate isomerase; GGPS, geranylgeranyl diphosphate synthase; PSY1, phytoene synthase; PDS, phytoene desaturase; ZISO, ζ-carotene isomerase; ZDS, ζ-carotene desaturase; CRTISO, carotene isomerase; LCYB, lycopene β-cyclase; CYCB, chromoplast-specific lycopene β-cyclase; LCYE, lycopene ε-cyclase; BCH, β-carotene hydroxylase; ECH, ε-carotene hydroxylase; ZEP, zeaxanthin epoxidase; VDE, violaxanthin de-epoxidase; NSY, neoxanthin synthase; NCED 9-cis-epoxycarotenoid dioxygenase. The metabolites are: pyruvate; GAP, D-glyceraldehyde 3-phosphate; HMBPP, (E)-4-hydroxy-3-methylbut-2-enyl diphosphate; DMAPP, dimethylallyl pyrophosphate; IPP, isopentenyl pyrophosphate; GGPP, geranylgeranyl diphosphate; Phytoene; ζ – carotene; lycopene; α – carotene; β – carotene; δ - carotene; lutein; β-cryptoxanthin; zeaxanthin; antheraxanthin; violaxanthin; 9-cis-neoxanthin; xanthoxin; ABA, abscisic acid (Figure is modified from [10, 11, 13, 16, 17])

Carotenoids are formed from isopentenyl diphosphate (IPP), a five-carbon compound, and dimethylallyl diphosphate (DMAPP), its allylic isomer. These compounds form geranylgeranyl diphosphate (GGPP) which in turn forms phytoene through the activity of *phytoene synthase (PSY)*. Phytoene forms lycopene via four desaturation reactions with the involvement of *phytoene desaturase (PDS) ζ-carotene isomerase (ZISO), ζ-carotene desaturase (ZDS)* and *carotene isomerase (CRTISO)* [13]. Lycopene, in turn undergoes a series of reactions to form lutein, through the ε, β-branch, the predominant carotenoid pigment in green plants [13], and violaxanthin from zeaxanthin with the presence of *zeaxanthin epoxidase (ZEP)* though the β, β-branch (Fig. 1). This forms the xanthophyll cycle, the mechanism that enables plant adaptation to high light stress [10]. 9-cis-neoxanthin is derived from the conversion of violaxanthin by *neoxanthin synthase (NSY)*, which in turn forms the phytohormone abscisic acid through *9-cis-epoxycarotenoid dioxygenase (NCED)* activity [11, 14], which controls abiotic stress signaling pathways [14].

The carotenoid biosynthetic pathway is controlled by the presence of the key enzyme PSY (Fig. 1, [10, 11, 13, 15–17]). Regarding loquat fruit, Fu et al. [10] investigated the mechanism underlying the differentiation of carotenoids in a red-fleshed (cv. ‘Luoyangqing’) and a white-fleshed (cv. ‘Baisha’) cultivar; differences in carotenoid accumulation in the two cultivars were linked with the differential expression of *PSYI*, *CYCB,* and *BCH* genes. The aim of the current study was to monitor the carotenoid composition in peel and flesh tissue of ‘Obusa’ fruits, an orange-fleshed cultivar, in correlation with the progress of fruit maturity. Towards this aim, high-resolution temporal expression profiles of carotenoid biosynthetic genes in both tissues were determined by RT-qPCR and linked with individual carotenoids, quantified by LC-MS.

## Methods

### Fruit material and experimental design

**Fig. 2:**
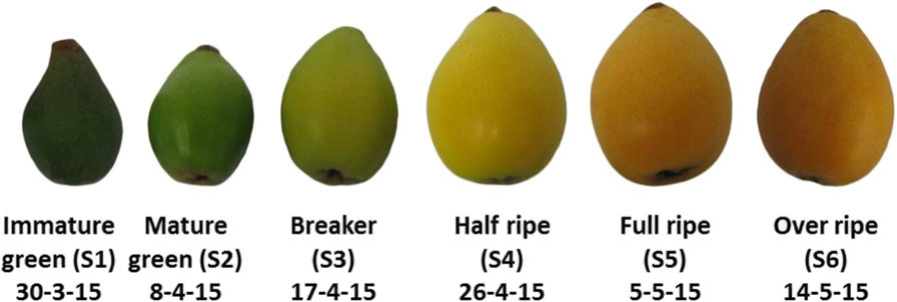
Phenotypes of loquat fruits during six distinct on-tree developmental stages (S1-S6)

Loquat fruits cv. ‘Obusa’ were harvested at ca. 10-day intervals, between March 30th and May 14th (Fig. 2), from a commercial orchard (Episkopi, Lemesos, Cyprus), owned by the first author. For each developmental stage, 30 uniform fruits were selected based on size and colour; such fruit were divided into three 10-fruit sublots, representing the biological replications. Fruit were initially used for the determination of physical dimensions and colour and subsequently for molecular and analytical analysis, as described below. The developmental stages were defined using the BBCH scale [2].

For the molecular analysis, fresh samples of both peel and flesh were flash frozen in liquid nitrogen and maintained at −80 °C until needed. For the determination of carotenoid profiles, samples were freeze-dried (Freeze Dryer-Christ Alpha 1–4 LD plus).

### Quality attributes

Physical dimensions of fruit were determined with the employment of an analytical grader and an electronic calibre (IS11112, Insize). The colour parameters CIE L* (brightness or lightness; 0 = black, 100 = white), a* (−a* = greenness, +a* = redness) and b* (−b* = blueness, +b* = yellowness) were measured at the peel and at the flesh tissue per fruit with a chroma meter (CR-400, Konica Minolta).

### Spectrophotometric determination of carotenoid and chlorophyll content

Twenty mL of acetone–hexane (4:6, *v*/v) were added to 100 mg of lyophilised plant material and thoroughly mixed. Upon separation of the two phases, the absorbance was determined in the supernatant at 453, 505, 645 and 663 nm. Based on Nagata and Yamashita equations, total carotenoids, chlorophyll-a and chlorophyll-b contents were determined [18].

### Liquid chromatography mass spectrometry (LC-MS) analysis of carotenoids

#### Extraction and saponification of loquat carotenoids

Liquid-liquid extraction and saponification of the samples was carried out as previously described by Minguez-Mosquera & Hornero-Mendez [19]. Briefly, 0.5 g of freeze-dried tissue (peel or flesh) was homogenized in acetone-butylated hydroxytoluene - 0.1% using an UltraTurrax (Ika, Staufen, Germany) and centrifuged (2000 rpm, 10 min, 4 °C). Extraction steps were repeated until complete removal of colour in the sample. The internal standard used was β-Apo-8′-carotenal (Sigma, St Louis, MO, USA). The extracts were combined and treated with diethyl ether. A solution of NaCl (10%, *w*/*v*) was added to separate the phases. The lower phase was discarded and the remaining phase was washed with Na_2_SO_4_ (2%, *w*/*v*) to remove water residues. Fifty mL of a methanolic solution of KOH (20%, *w*/*v*) was added and left for 1 h in darkness. The organic phase was washed several times with deionized water until washings were neutral. It was then filtered through a bed of anhydrous Na_2_SO_4_ and evaporated until dry using a speed vacuum (Thermo Scientific Savant SPD121P). The pigments were collected with 1 mL of acetone: methanol (7:3, *v*/v) and stored at −20 °C until needed. To prevent isomerization and photodegradation of carotenoids, all procedures were carried out under pale light.

#### LC- MS analysis of loquat carotenoids

The carotenoid analysis was carried out using an Agilent 1200 HPLC equipped with a photodiode array detector and a single quadrupole mass spectrometer detector in series (6120 Quadrupole, Agilent Technologies, Santa Clara, CA, USA). Chromatographic separation was performed on a reverse-phase Poroshell 120 EC-C_18_ column (100 mm × 3 mm, 2.7 μm particle size) (Agilent Technologies) operating at 32 °C. Water with 0.05 M ammonium acetate and acetonitrile: methanol (70:30) were used as mobile phases A and B, respectively, with a flow rate of 0.7 mL min^−1^. The linear gradient started with 60% of solvent B in A, reaching 100% solvent B at 20 min; this was maintained up to 35 min. The initial conditions were re-established at 36 min and kept under isocratic conditions up to 40 min. Injection volume was 5 μL. Detection and quantification of all carotenoids and carotenoid esters were carried out in UV-vis at 450 nm (Additional file 1: Figure S1).

The identification of carotenoids in loquat was carried out on an Agilent 1100 HPLC system equipped with a photodiode array and an ion trap mass spectrometer detector (Agilent Technologies, Waldbronn, Germany). The mass detector was a Bruker ion trap spectrometer (model HCT Ultra) equipped with an APCI (Atmospheric Pressure Chemical Ionization). The mass spectrometer parameters were as follows: positive ion mode (APCI +); source temperature, 350 °C; probe temperature, 450 °C, corona voltage, 4.0 kV; The full scan mass covered the range from *m/z* 100 up to *m/z* 1200 and the target mass was adjusted to 350. Collision-induced fragmentation experiments were performed in the ion trap using helium as the collision gas, with voltage ramping cycles from 0.3 up to 2 V. Mass spectrometry data were acquired in the positive mode, and the MS^n^ was carried out in the automatic mode. The identification of the peaks was performed by the extracted ion-chromatograms of the ion current at *m/z* values corresponding to the [M-H]^+^ ions of the individually investigated compounds, as well as their fragmentation. Furthermore, to confirm the identification of the carotenoids and obtain a more reliable identification, samples were analyzed with the Agilent 1290 Infinity UPLC system coupled to a quadrupole (Q-TOF) mass spectrometer (6550 Accurate-Mass QTOF, Agilent Technologies) using an electrospray interface with jet stream technology. The chromatographic separation was developed under the same conditions, as described above. The optimal conditions for the electrospray interface were as follows: gas temperature: 300 °C, drying gas 11 L/min, nebulizer 65 psi, sheath gas temperature 400 °C, sheath gas flow 12 L/min. Spectra were acquired in the *m/z* range of 100–1100, in a positive mode and with an acquisition rate of 1.5 spectra in MS, maintaining a mass resolution over 50,000 for the mass range used. Internal mass calibration by the simultaneous acquisition of reference ions and mass drift compensation was used for obtaining low mass errors. Q-TOF MS data were processed using the Mass Hunter Qualitative Analysis software (version B.06.00). For quantification, β-apo-8′-carotenal was used as an internal standard. Lutein, β-carotene and violaxanthin (Sigma, St Louis, MO, USA) in a concentration range of 5–100 μg.mL^−1^ were used to quantify compounds in three different groups, hydroxycarotenoids, carotenes, and epoxycarotenoids, respectively. Neoxanthin, neochrome, β-cryptoxanthin epoxides and β-carotene epoxides were estimated as violaxanthin. β-Cryptoxanthin was quantitated as lutein. The *cis*-isomers were quantitated with the calibration curve of the all-*trans* isomers. Concentrations were expressed as micrograms of pigment per 100 g of sample fresh weight (μg 100 g^−1^ F.W.).

### RNA extraction, cDNA synthesis and quantitative real-time RT-PCR analysis

Total RNA was extracted from three bulked biological replicates of loquat fruit material for each developmental stage (S1-S6) according to a modified cetyltrimethylammonium bromide (CTAB) protocol developed by Gambino et al. [20]. Next, RNA integrity was confirmed spectrophotometrically (Nanodrop 1000 Spectrophotometer, Thermo Scientific) followed by gel electrophoresis and samples were treated with RNase-free DNase (Cat. No. NU01a, HT Biotechnology LTD, England) to remove total gDNA, as elsewhere described [21].

Total RNA (0.5 μg) was reverse transcribed using the PrimeScript™ RT reagent Kit (Takara Bio, Japan), according to the manufacturer’s protocol (Takara Bio, Japan). Subsequently, quantitative real-time RT-PCR was performed with BioRad IQ5 real-time PCR cycler (BioRad, USA). In total, three biological replicates were analyzed for each developmental stage for both loquat peel and flesh. The reaction mixture consisted of 4 μL cDNA in reaction buffer (15-fold diluted first-strand cDNA for all genes except for *VDE* and *LCYE* that were diluted 5-fold), 0.5 μL of each primer (10 pmol/μL) and 5 μL SensiFAST™ SYBR^®^ & Fluorescein mix 2× (Bioline). The total reaction volume was 10 μL. The initial denaturation step was at 95 °C for 5 min, followed by 40 cycles of amplification [95 °C for 30 s, annealing temperature (Ta ^o^C) for 30 s, and 72 °C for 30 s] and a final elongation stage at 72 °C for 5 min. Gene amplification cycle was followed by a melting curve run, carrying out 61 cycles with 0.5 °C increment from 65 to 95 °C. The annealing temperature of previously published primers for loquat carotenoid biosynthetic genes (58 to 65 °C) is shown in Additional file 1: Table S1. Loquat’s *actin* gene was used as a housekeeping reference gene (*EjACT)*.

### Statistical analyses

Statistical analyses were carried out by comparing the averages of each developmental stage based on the analysis of variance (one way ANOVA) according to Duncan’s multiple way test with a significance level of 5% (*P* ≤ 0,05), using the SPSS v.17.0 statistical analysis software package.

The relative quantification and statistical analysis of gene expression levels was performed with the REST-XL software, using the pairwise fixed reallocation randomization test [22]. Gene expression levels were normalized against the *EjACT* housekeeping reference gene; the initial developmental stage (S1) both for peel and flesh tissue was used for calibration.

## Results and discussion

### Qualitative attributes

Fruit weight, length and width ranged between 25.3–59.1 g, 48.8–57.0 mm and 34.4–45.0 mm, respectively (Additional file 1: Table S2). Maximum fruit size and weight was recorded at stage S5, which coincides with the optimum maturity stage for harvest.

Colour parameters in the flesh ranged between 52.12–74.32 for L*, −11.51-10.67 for a* and 30.75–36.67 for b*. The corresponding values for L^*^, a^*^, b^*^ parameters in the peel were 45.65–64.99, −17.66-12.42 and 28.20–49.19 (Additional file 1: Table S3). Previous study in loquat cultivars indicated that the a*/b* chroma ratio receive negative values in immature fruits, around zero for pale yellow-colored fruits, and positive values for orange-colored loquat fruits; thus higher ratio can be linked with higher carotenoid accumulation [1]. In our study, the a*/b* ratio in the flesh received negative values at stages S1*-*S3, implying the green colour, the S4 value indicates the colour break and the S5 and S6 had the higher values. Similarly, the a*/b* ratio was lower in the peel during the first three early stages (−0.60 to −0.20), close to zero in the breaker stage, while it received positive values during the last maturation stages (0.24–0.29) (Additional file 1: Table S3). Overall, the a*/b* ratio can be linked with total carotenoid accumulation and its transient increase with the progress of on-tree development, in accordance with previous studies [1, 16].

### Carotenoid and chlorophyll contents

Initially, a rapid colorimetric assay was employed to screen carotenoid and chlorophyll contents. Total carotenoids varied between 0.8–6.1 and 0.1–5.9 mg 100 g^−1^ FW β-carotene equivalents in the peel and in the flesh, respectively (data not shown). The highest concentrations in Chl a and Chl b were found in the peel tissue at the immature green stage (18.0 and 12.1 mg 100 g^−1^ FW, respectively), while their contents were substantially lower in the flesh (0.6 and 0.5 mg 100 g^−1^ FW at S1 stage, respectively) and degraded thereafter with the progress of on-tree ripening. During ripening of fleshy fruits chloroplasts turn into chromoplasts; this process encompass a transient increase of carotenoids and degradation of chlorophylls [23]. Chl a is a blue-green coloured pigment and is less stable than the yellow-green Chl b.

### Identification of carotenoids in loquat fruit using LC-MS

Carotenoids in the peel and in the flesh were identified and quantified (Table 1, Additional file 1: Figure S1). Thirty-two carotenoids were detected by HPLC-DAD and LC-MS techniques. Peak identification was based on their relative retention time values, their UV-Vis spectra, their mass spectra, information from the literature and comparison with authentic standards when possible. Table 1 summarizes the identification data for each carotenoid, including chromatographic and spectroscopic values.

**Table 1:** UV/vis spectra and characteristic ions of carotenoids from six maturation stages of loquat fruits, obtained by HPLC-PDA-MS

Peak	Carotenoid	t_R_ (min)	λ_max_ (nm)	%III/II	%Ab/II	[M + H] + m/z	HPLC/APCI (+)-MS^n^ experiment *m/z* (% base peak)	Exact mass	Score	Error (ppm)	Molecular formula
1	All-*trans*-neoxanthin	14.1	412,436,462	70	0	601 (40), 583 (75), 565 (100)	MS^2^ [601]: 583 (100), 565 (47), 547 (9), 509 (6),491 (5), 221 (41) MS^3^ [601˃583]: 565 (48), 547 (14)	600.4188	91.88	-2.69	C_40_H_56_O_4_
2	All-*trans*-neochrome	14.6	397,420,448	90	0	601 (42), 583 (100), 565 (43)	MS^2^ [601]: 583 (100), 565 (57), MS^3^ [601˃583]:221 (62)	600.4178	96.70	-0.89	C_40_H_56_O_4_
3	All-*trans*-violaxanthin	14.8	414,438,470	75	0	601 (85), 583 (100), 565 (20)	MS^2^ [601]: 583 (100), 565 (12), 509 (5), 221 (24)	600.4178			C_40_H_56_O_4_
4	Not identified	16.0	466	0	0	455 (100)	MS^2^ [455]: 437 (80), 399 (34)	455.3324	93.60	-2.61	C_33_H_43_O
5	Not identified	16.2	396,420,448	75	0	601 (60)	MS^2^ [601]: 583 (95), 221 (100				
6	Not identified	16.4	Not detected	--	--	601 (64)	MS^2^ [601]: 583 (90), 565 (40), 491 (9), 221 (100) MS^3^ [601˃583]:565 (100),221 (60)				
7	β- Diepoxy-cryptoxanthin	16.7	412, 436,466	72	0	585 (100)	MS^2^ [585]: 567 (45), 549 (80), 493 (37), 221 (100), 205 (10)	584.4184	95.60	-2.52	C_40_H_56_O_3_
8	*Cis*-violaxanthin	17.3	324,410, 434,464	60	8	601 (81), 487 (100)	MS^2^ [601]: 583 (100), 565 (42), 509 (6),491 (23), 221 (51) MS^2^ [487]: 469 (100)				
9	Not identified	17.8	378,400,424	100	0	601 (100), 351 (98)	MS^2^ [601]: 583 (100), 565 (13), 509 (15), 491 (14),393 (40),221 (41) MS^2^ [351]: 333 (16)				
10	All-*trans*-lutein	18.2	420,444,472	48	0	569 (5), 551 (100)	MS^2^[551]: 533 (51), 495 (24), 477 (35)	568.427	99.30	-0.226	C_40_H_56_O_2_
11	Not identified	18.5	444	0	0	454 (100)	MS^2^[454]: 436 (7), 393 (100)				
12	Not identified	18.9	448	0	0	473 (100),539 (80)	MS^2^[539]: 521 (100)				
13	Not identified	19.0	454	0	0	473 (100), 454 (14)	MS^2^ [473]: 455 (43), 205 (100) MS^2^ [454]: 436 (31), 393 (36)				
14	Not identified	19.3	423,438,474	60		551 (100)	MS^2^ [551]: 533 (67), 477 (55)				
15	Not identified	19.4	448	0	0	439 (93), 403 (100)	MS^2^ [439]:403 (14)				
16	Not identified	19.6	434	0	0	391 (100)	MS^2^ [391]:373 (14)				
17	5,6-Epoxy-β-cryptoxanthin	19.8	416,438,466	34	0	569 (17),551 (54)	MS^2^ [551]: 533 (20), 205 (21)	568.428	75.56	-5.07	C_40_H_56_O_2_
18	5′,6′-Epoxy-β-cryptoxanthin	19.9	419,441,470	52	0	569 (30), 551 (100)	MS^2^ [551]: 533 (100), 577 (14), 459 (30), 221 (13)	568.428	89.42	-2.11	C_40_H_56_O_2_
19	*Cis*-lutein	20	326,412,436,462	52	20	569 (18), 551 (100)	MS^2^ [551]: 533 (100), 221 (11)	568.428	77.7	3.57	C_40_H_56_O_2_
32	β- Diepoxy-cryptoxanthin	20.2	416,440, 470	87	0	585 (100)	MS^2^ [583]: 567 (49), 549 (10) 221 (31), 205 (29)				
20	Not identified	20.4				446 (100) 417 (90)	MS^2^ [417]:399 (100) MS^2^ [446]:219 (100)				
21	β-Apo-8′-carotenal (IS)	21.0	450	0	0	417 (100)	MS^2^ [417]:399 (25), 389 (32), 361 (39), 325 (100), 293 (97), 157 (88), 119 (16)	416.3079	98.23	-2.15	C_30_H_40_O
22	Citranaxanthin	21.6	470	0	0	457 (100)	MS^2^ [457]:439 (90), 399 (49)	455.3324	97.23	-1.25	C_33_H_44_O
23	Not identified	22.4	470			696 (100)	MS^2^ [696]: 534 (34), 516 (100)				
24	Not identified	22.8	420,444,470	31	0	537 (58)	MS^2^ [537]: 467 (16), 444 (51), 365 (100)				
25	Not identified	23.3	Not detected			537 (28),430 (100)	MS^2^ [537]:444 (100),481 (24) 413 (90)				
26	All-*trans*-β-cryptoxanthin	24.0	420, 448,472	24	0	553 (100)	MS^2^ [553]:535 (100), 497 (41),461 (10)				
27	Not identified	24.5				664 (100)	MS^2^ [664]: 551 (100), 496 (55)				
28	Not identified	24.9	453, 479	0	0	551 (100)	MS^2^ [551]: 534 (15), 361 (100)				
29	Phytoene + not identified 14	28.0				545 (95), 553 (100)	MS^2^ [545]:489 (10),395 (100) MS^2^ [553]:535 (12)				
30	5,8-epoxy-β-carotene	28.4	405, 424, 450	26	0	553 (100)	MS^2^ [553]:535 (55),461 (35), 221 (64),205 (17)				
31	All-*trans*-β-carotene	36.5	424, 446,470	18	0	537	MS^2^ [537]:444 (100)				

All-*trans*-neoxanthin (peak 1) showed a characteristic UV-visible spectrum. The molecular mass of neoxanthin was confirmed by the protonated molecule at *m/z* 601 and by consecutive losses of three hydroxyl groups from the protonated molecule, at *m/z* 583, 565 and 547, verified by MS/MS. The UV-visible absorption spectrum of neochrome (peak 2) showed λmax at 397, 420 and 448 nm with high spectral fine structure (%III/II 90); these values are in agreement with previous studies in loquat [9]. All-trans-violaxanthin (peak 3) was identified by comparison with the authentic standard. The protonated molecule at *m/z* 601, and the fragments at *m/z* 583 and 565, due to the losses of hydroxyl groups and at *m/z* 221, all formed from 601 at both MS/MS and in-source fragmentations. Peak 8 was tentatively identified as *cis*-violaxanthin with a mass spectrum, lower λmax and spectral fine structure values similar to those of peak 3. β-diepoxy cryptoxanthin, peak 7 and 32 (only in flesh in S5 and S6)] showed the [M + H]^+^ at *m/z* 585. The second order MS experiments revealed a fragment at *m/z* 567 due to the loss of water and the ions at *m/z* 221 and at *m/z* 205 characteristic of the epoxide group with one ion located in a ring with a hydroxyl group and another one in an unsubstituted ring respectively. All *trans*-lutein was identified by comparison with an authentic standard. All *trans*-lutein (peak 10) and *cis*-lutein (peak 19) showed characteristic UV-visible spectra, with a hypsochromic shift of 8 nm for the *cis* isomer. The identification of both lutein isomers was confirmed by their mass spectra with the protonated molecule at *m/z* 569 and fragments at *m/z* 551 and *m/z* 533 due to the loss of one and two hydroxyl group respectively. The MS/MS showed, in addition, the presence of fragments at *m/z* 477 resulting from the loss of toluene ([M + H-92] ^+^) from the polyene chain. In APCI-MS, the fragment with *m/z* 551 presented a higher intensity than the protonated molecular ion (*m/z* 569). Peaks 17 and 18 were identified as mono-epoxides of β-cryptoxanthin considering their UV-vis and MS characteristics by the comparison with literature data [9, 24]. The mass spectra of both epoxides presented the protonated molecule at *m/z* 569 and fragment ion at *m/z* 551 due to the loss of a hydroxyl group. Peak 17 was designated as 5′,6′-epoxy- β-cryptoxanthin due to the presence of the mass fragment at *m/z* 205 that is consistent with the location of one epoxide group in the unsubstituted ring whilst peak 18 showed the mass fragment at *m/z* 221, indicating that the epoxide groups were in a ring with a hydroxyl group. β-Apo-8′-carotenal was identified as peak 21 (internal standard). The mass spectra presented the protonated molecule at *m/z* 417. Ions of *m/z* 399 and 389 were detected corresponding to the loss of water and carbon monoxide respectively. Elimination of toluene from the protonated molecule was observed at *m/z* 325. The use of an internal standard was recommended to estimate the losses during the extraction process. Saponification with potassium hydroxide has been an integral part of carotenoid analyses. Kimura et al. [25] showed that β-apo-8′-carotenal was completely transformed to citranaxanthin (peak 22), apparently by aldol condensation with acetone. The conversion percentage from β-apo-8′-carotenal to citranaxanthin was 98% and their sum was considered for quantification. The identification of citranaxanthin was confirmed on the basis of its protonated molecule at *m/z* 457 [26] and its characteristic UV-vis spectrum [27]. Due to the presence of the same chromophore, β-cryptoxanthin (peak 26) and β-carotene have similar UV-visible spectra. As expected, the protonated molecule was detected at *m/z* 553 and the MS/MS revealed the presence of fragment ions at *m/z* 551 and 461 corresponding to the losses of the hydroxyl group and toluene. 5,8-epoxy-β-carotene (peak 30) could not be identified by its UV-visible spectral characteristics. Mass spectra highlighted the protonated molecule at *m/z* 553 and the MS/MS showed the presence of fragment ions at *m/z* 551 and 461 corresponding to the losses of the hydroxyl group and toluene, respectively, and at *m/z* 221 that corresponds to the location of the epoxide group in the 3-hydroxy-β-ring. The mass spectra of beta-carotene, peak 31, showed the protonated molecule at *m/z* 537 and a fragment ion in the MS/MS at *m/z* 444, corresponding to the loss of the toluene from the polyene chain.

### Carotenoid composition in loquat fruit

**Fig. 3:**
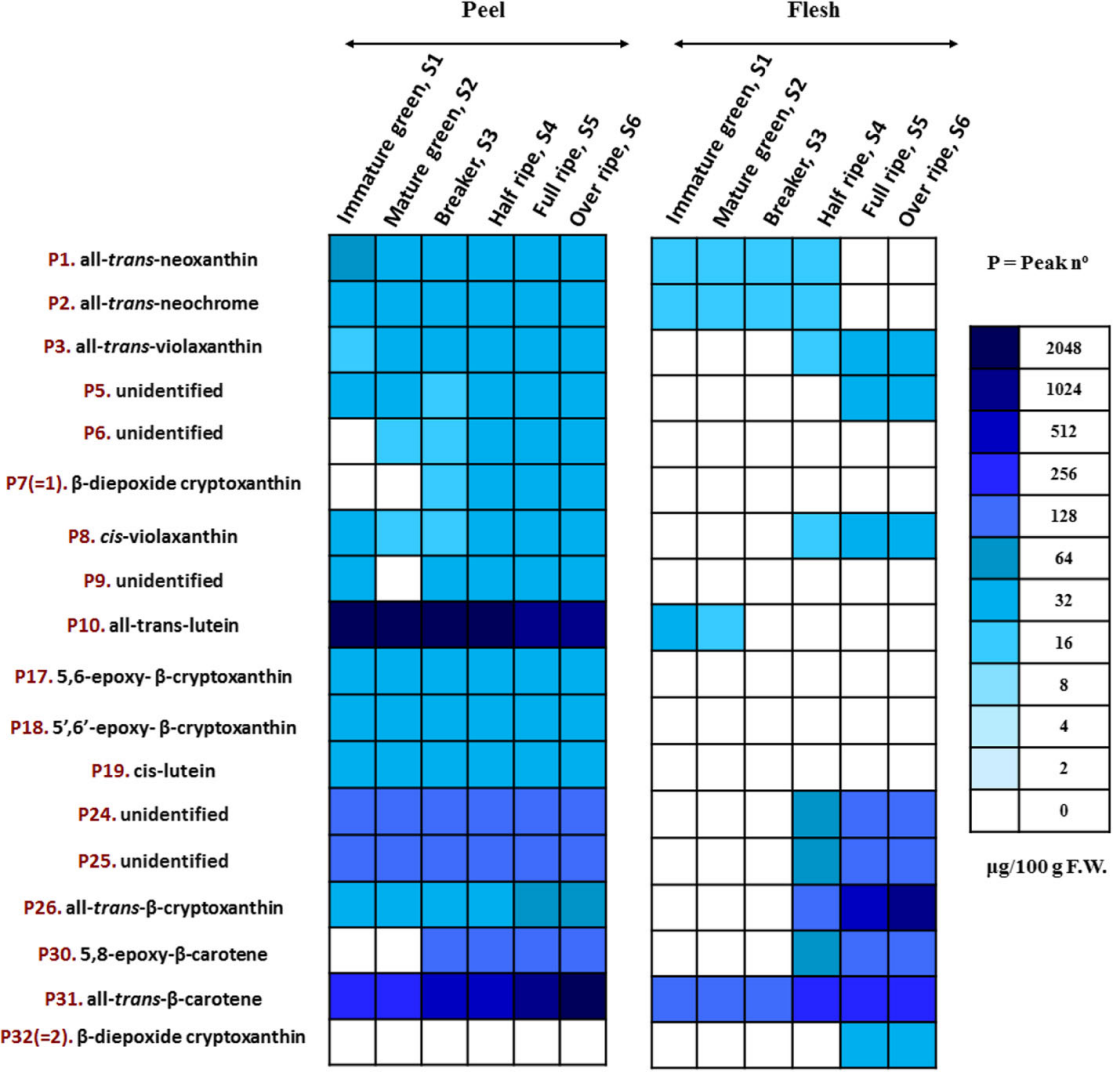
Heat map of the quantification of the identified carotenoids (Τable 1) in the peel and in the flesh of loquat fruit (cv. ‘Obusa’) during six on-tree developmental stages (*n* = 3). Results are expressed as μg 100 g^−1^ fresh weight (F.W.). A scale of colour intensity is presented as a *legend*. Actual quantification values of the identified carotenoids are shown in Additional file 1: Table S4

Results revealed great differences in carotenoid composition between peel and flesh. In particular, 32 carotenoids were found in loquat peel, while only eighteen carotenoids were identified in the flesh. Except for qualitative differences, the concentration of carotenoids was significantly higher in the peel than in the flesh. This was not the case when total carotenoids were determined spectrophotometrically, indicating the limitations of such colorimetric assays. Chromatograms also revealed that the major carotenoids in peel were *trans*-lutein and *trans*-β-carotene. The concentration of *trans*-lutein decreased with the progress of ripening from 1621.5 to 688.4 μg 100 g^−1^ FW. On the other hand, *trans*-β-carotene content increased drastically from 151.9 to 1096.9 μg 100 g^−1^ FW. The biosynthesis of some carotenoids such as *trans*-β-cryptoxanthin, 5,8-epoxy-β-carotene, β-diepoxy-cryptoxanthin and *cis*-violaxanthin has also been monitored (Fig. 3, Additional file 1: Table S4). Conversely, *trans*-neoxanthin and *trans*-neochrome decreased or remained stable with the progress of on-tree fruit development in the peel, while they were not detected in the flesh during the last developmental stages.

The carotenoid profiling of loquat flesh was found to be quite different from the peel. The most abundant carotenoid in mature fruits was *trans*-β-cryptoxanthin, followed by *trans*-β-carotene, compounds 18 and 31, and 5,8-epoxy-β-carotene (peak 30) (Table 1, Fig. 3, Additional file 1: Table S4). An increment in the concentration of all carotenoids during on-tree development except for *trans*-neoxanthin, *trans*-neochrome and *trans*-lutein was found (Fig. 3, Additional file 1: Table S4). Overall, a great effect of the developmental stage on the carotenoid composition was revealed.

All-*trans*-neochrome, all-*trans*-violaxanthin, β-diepoxy-cryptoxanthin, *cis*-violaxanthin, all-*trans*-lutein, 5,6-epoxy-β-cryptoxanthin, 5′,6′-epoxy-β-cryptoxanthin, all-*trans*-β-cryptoxanthin, phytoene and all-*trans*-β-carotene were previously identified in five loquat cultivars, originating from Brazil [9]. In their findings, they reported *trans*-β-carotene (19–55%), *trans*-β-cryptoxanthin (18–28%), 5,6:5,6 -diepoxy-β-cryptoxanthin (9–18%) and 5,6-epoxy-β-cryptoxanthin (7–10%) to be the main carotenoids. In the flesh, it was found that β-carotene and lutein were the major carotenoids with neoxanthin, violaxanthin, luteoxanthin, 9-*cis*-violaxanthin, phytoene, phytofluene and ζ–carotene also present.

The carotenoid quantification in our study proved that the peel had higher carotenoid concentrations than the flesh, except for β-diepoxy-cryptoxanthin which was found from the 3rd until the 6th maturity stage in the peel, ranging from 15.0 ± 0.8 to 21.2 ± 1.7 μg 100 g^−1^ FW, as well as in flesh during the last maturity stages (S5 and S6) at 17.6 ± 1.2 and 21.4 ± 1.8 μg 100 g^−1^ FW, respectively (Fig. 3, Additional file 1: Table S4). *Trans*-neoxanthin and *trans*-neochrome appeared throughout all developmental stages in the peel as well as in the first 4 maturation stages in the flesh. On the other hand, *trans*-violaxanthin increased in the peel with the progress of on-tree ripening (15.6 ± 2.5 to 25.2 ± 4.6 μg 100 g^−1^ FW), while it was detected in the flesh during the last stages (S4-S6) with progressive increase (from 12.5 ± 0.2 to 21.5 ± 1.6 μg 100 g^−1^ FW). Similar findings and trend were observed for *cis*-violaxanthin which ranged from 16.2 ± 2.5 to 23.2 ± 3.0 μg 100 g^−1^ FW in the peel, being detectable in the flesh from S4 stage onwards (12.3 ± 0.1 to 19.1 ± 1.5 μg 100 g^−1^ FW). 5′,6′-Epoxy-β-cryptoxanthin and *cis*-lutein were detected exclusively in the peel throughout all developmental stages (Fig. 3). Intriguingly, *trans*-lutein in the peel registered the highest contents during the initial developmental stages and went descending thereafter, meanwhile it was found in detectable amounts in the flesh only at stages 1 and 2, yet substantially lower compared to the peel. Citranaxanthin and phytoene have also been identified, although they were not quantified (Table 1).

### Gene expression profiles

**Fig. 4:**
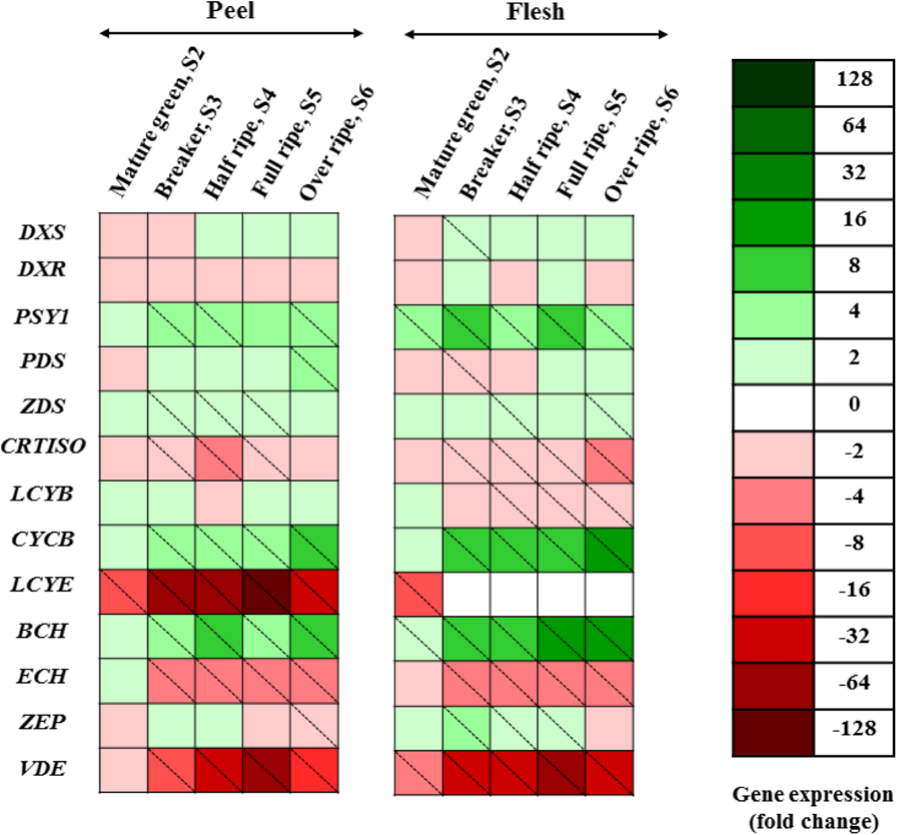
Heat map of the relative expression levels of carotenoid biosynthesis genes (*DXS, DXR, PSY1, PDS, ZDS, CRTISO, LCYB, CYCB, LCYE, BCH, ECH, ZEP* and *VDE*) in loquat fruit (cv. ‘Obusa’), both in the peel and in the flesh, during five on-tree developmental stages (S2-S6) (*n* = 3). Relative mRNA abundance was evaluated by real-time RT-PCR using three biological repeats. Up-regulation is indicated in *green*; down-regulation is indicated in *red*. A *diagonal line in a box* indicates a statistically significant value (*P* ≤ 0.05). A scale of colour intensity is presented as a *legend*. The first developmental stage (S1) both for the peel and the flesh was used for calibrating gene exeression values. Actual relative expression levels are shown in Additional file 1: Tables S5-S6 and Figs. S2-S3

With the aim to shed light on the carotenoid biosynthetic pathway in loquat fruit, the expression profile of thirteen known genes of the carotenoid pathway was analyzed, showing differential expression patterns in the peel and the flesh tissue (Fig. 4, Additional file 1: Tables S5–S6 and Figures S2–S3). For gene expression analyses, each tissue was examined individually, considering S1 stage as the calibrator of the tissue tested (peel or flesh).

Middle and downstream genes of the carotenoid biosynthetic pathway (*CYCB, LCYE, BCH, ECH* and *VDE*) showed clear differentiation in their expression levels among S2-S6 developmental stages compared with the remaining genes. In both peel and flesh, *PSY1*, *ZDS*, *CYCB,* and *BCH* were significantly up-regulated in most of the developmental stages compared with the S1 stage respectively, whereas *CRTISO*, *LCYE*, *ECH* and *VDE* were generally down-regulated. *PDS* was significantly up-regulated at the S6 stage in the peel, while it was significantly suppressed at the S3 stage in the flesh tissue. *ZDS* presented an increase in expression levels between the S3 and S5 stage in peel, though the increase was at the S4 and S6 stage in flesh tissue. Non statistically significant changes in expression levels of *DXS, DXR* (both involved in MEP pathway) and *LCYB* were monitored in the peel in comparison with the S1 stage; in flesh *DXR* followed the same trend whereas, *DXS* presented an accumulation at the S3 and *LCYB* transcript levels decreased gradually through stages S4-S6 which could account for the low or non-detectable *trans*-lutein findings in the flesh (Figs. 1, 3 and 4). *ZEP* expression levels presented highest suppression at the S6 stage in peel, contrarily to flesh profile where it was up-regulated at the S3-S5 stages.

*BCH* and *CYCB* transcript levels were substantially up-regulated with the progress of on-tree fruit development both in the peel and in the flesh, registering the highest values at the last stage for both tissues (Fig. 4, Additional file 1: Tables S5, S6). Notably in flesh tissue, a 8.1- and 11.0- fold increase of *CYCB* and *BCH* transcripts during the last stage compared to the initial stage was recorded, respectively. *PSY1* presented statistically significant increases at the S3, S4 and S6 stages of the peel and throughout all stages in the flesh (S2-S6); the most prominent gene regulation in the flesh was found at the S3 and S5 stages (Additional file 1: Table S6).

Contrarily, *CRTISO, LCYE, ECH and VDE* demonstrated overall a down-regulation expression pattern in both peel and flesh tissue. *CRTISO* was down-regulated at stages S3-S5 in peel; a similar trend was monitored in the flesh (S3-S6 stages). *ECH* had similar expression pattern (suppression) for both peel and flesh from stage S3 onwards. *LCYE* was down-regulated in peel throughout the five developmental stages; the most abundant decrease (128 fold change) was registered at the S5 stage. Interestingly, in flesh the decrease was registered at the S2 stage and remained undetected thereafter. *VDE* expression levels were repressed throughout S3 to S6 stages in peel and throughout all developmental stages in flesh. In both peel and flesh, *VDE* presented a substantial suppression at the S5 stages (36.8 and 49.5 fold change, respectively) compared with S1 stage.

Notably, *PSY1*, known to catalyse the first step in the carotenoid formation [14], expression levels depicted a general up-regulation with the progress of on-tree fruit development both in the peel and in the flesh; however the highest transcript values in flesh tissue were monitored at S3 and S5 stages, not concomitant with total carotenoid accumulation. In another fleshy fruit (apple), Ampomah-Dwamena et al. [28] also postulated that *PSY1* expression levels had no direct correlation with carotenoid content in different genotypes. On the other hand, *PDS*, an upstream pathway gene, presented statistically significantly higher transcript levels only in the peel at the last developmental stage (S6); *PDS* expression levels have been correlated with high- and low- carotenoid content apple cultivars [28].

*Trans*-lutein presented appreciably high accumulation in the first four stages in the peel with a reduction at ripe and over-ripe stages, while detectable amounts in the flesh were registered only during the initial developmental stages, as chloroplasts began to develop into chromoplasts. This decrease can be attributed to the fact that (1) *LCYE* was markedly down-regulated throughout the developmental stages in both peel and flesh compared to corresponding per tissue immature stage (notably not detectable transcripts during S3-S6 of flesh was monitored), (2) *LCYB* was down-regulated over the last developmental stages in flesh (S4-S6) and (3) *ECH* mRNA expression was generally down-regulated both in the peel and in the flesh (S3-S6). Fu et al. [10] noted that lutein is showing a transient decrease in the flesh of loquat cultivars with the progress of on-tree fruit development, whereas there is no connection with *BCH* expression which appears to be up-regulated especially in the red-fleshed cultivar ‘Luoyangqing’. An up-regulation of *BCH* gene expression was also monitored in our study in a similar flesh-type loquat cultivar. Ampomah-Dwamena et al. [28] showed a close correlation between *LCYE* expression and carotenoid content in apple fruit skin. This was not the case with the flesh; suggesting that down-regulation of *LCYE* is consistent with lower *trans*-lutein concentrations in the flesh.

Contrarily, the high *BCH* expression values registered in the flesh at the last two stages can be linked with the transient carotenoid accumulation of *trans*-β-cryptoxanthin in these stages, concomitant with higher mRNA expression of *CYCB* and non-detectable *LCYE* transcripts (Stages S3–S6). These findings are in accordance with Fu et al. [10], where the *BCH* values for the red-fleshed cultivar ‘Luoyangqing’ showed a transient increase at the breaker stage. In addition, *CYCB* expression levels were also higher after the S4 stage. Kato et al. [11] stated that the decrease of *LCYE* gene expression in Citrus fruits is related with higher contents of β-carotene as the ε, β-branch of the carotenoid pathway shifts to the β, β-branch during transition from immature to mature stage. Zhao et al. [16] found that *BCH* is responsible for high β-cryptoxanthin content in persimmon fruit, in accordance with findings in other loquat cultivars [29]. The latter study suggests a direct link between the synthesis and accumulation of β-cryptoxanthin and the abundant expression of *BCH*. The transient increase of *trans*-β-carotene towards the S6 peel stage can also be linked with the up-regulation of *CYCB* and down-regulation of *LCYE,* as elsewhere described [10]. Zhang et al. [29] also links the higher β-carotene level in loquat peel with the abundant increase of *PSY1*, as well as *CYCB* and *BCH* mRNA expression levels.

*VDE* expression which leads to violaxanthin biosynthesis is significantly suppressed in almost all stages, both in the flesh and in the peel compared with the calibrator (S1 stage) (thus expecting very little violaxanthin); in the case of *ZEP*, which converts violaxanthin back to precursor molecules such as zeaxanthin, the main trend is that it is induced in several stages in the flesh. This is in accordance with metabolite levels, as both *cis*- and *trans-*violaxanthin are at appreciable low concentrations and/or non-detectable during several stages in the flesh (Figs. 3, 4; Additional file 1: Table S4).

## Conclusions

The carotenoid profile of ‘Obusa’ fruits, an orange-fleshed loquat cultivar, was elucidated during distinct on-tree developmental stages. Results indicated that carotenoid composition was greatly affected during fruit development, revealing evident differentiations between flesh and peel tissue. The major carotenoids were *trans*-lutein and *trans*-β-carotene in the peel, and *trans*-β-cryptoxanthin, *trans*-β-carotene, and 5,8-epoxy-β-carotene in the flesh. To the best of our knowledge, the presence of *cis*-lutein, citranaxanthin and 5,8-epoxy-β-carotene has not been reported previously in loquat, but only in other fruits of tropical origin [30, 31]. Furthermore, a link was attempted to be established between gene up- or down-regulation during the developmental stages of the loquat fruit, and how their expression affects carotenoid content. Elevated content of *trans*-β-carotene both in the flesh and in the peel with the progress of on-tree fruit development can be linked with the up-regulation of *CYCB,* a main carotenoid biosynthetic gene. Notably, the non-detectable amounts of *trans*-lutein in the flesh during the S3-S6 stages can be linked with the significant suppression of *LCYB* and *LCYE* expression levels during these stages. Transcripts levels of the latter gene were also significantly reduced throughout all developmental stages in the peel compared with the immature stage.

## Additional file

Additional file 1: Figures S1-S3 and Tables S1-S6 illustrate extra information cited in the text. (ZIP 182 kb)

## Abbreviations

ABA: Abscisic acid
*BCH*: *β-carotene hydroxylase*
Chl a: Chlorophyll-a
Chl b: Chlorophyll-b
*CRTISO*: *Carotene isomerase*
*CYCB*: *Chromoplast-specific lycopene β-cyclase*
DMAPP: Dimethylallyl pyrophosphate
*DXR*: *DXP reductoisomerase*
*DXS*: *1-deoxy-D-xylulose 5-phosphate-synthase*
*ECH*: *ε-carotene hydroxylase*
*EjACT*: *Eriobotrya japonica Actin*
FW: Fresh weight
GAP: D-glyceraldehyde 3-phosphate
GGPP: Geranylgeranyl diphosphate
*GGPS*: *Geranylgeranyl diphosphate synthase*
HMBPP: (E)-4-hydroxy-3-methylbut-2-enyl diphosphate
*IDI*: *Isopentenyl pyrophosphate isomerase*
*IDS*: *Isopentenyl pyrophosphate synthase*
IPP: Isopentenyl pyrophosphate
*LCYB*: *Lycopene β-cyclase*
*LCYE*: *Lycopene ε-cyclase*
*NCED*: *9-cis-epoxycarotenoid dioxygenase*
*NSY*: *Neoxanthin synthase*
*PDS*: *Phytoene desaturase*
*PSY1*: *Phytoene synthase*
*VDE*: *Violaxanthin de-epoxidase*
*ZDS*: *f-carotene desaturase*
*ZEP*: *Zeaxanthin epoxidase*
*ZISO*: *ζ-carotene isomerase*
