# Supplementary material for: Metabolic and transcriptional elucidation of the carotenoid biosynthesis pathway in peel and flesh tissue of loquat fruit during on-tree development
Source: BMC Plant Biol. 2017 Jun 14;17:102. doi: 10.1186/s12870-017-1041-3 (PMC5471668; doi:10.1186/s12870-017-1041-3)
Supplement: Additional file 1: — Figures S1-S3 and Tables S1-S6 illustrate extra information cited in the text. (ZIP 182 kb) [file 12870_2017_1041_MOESM1_ESM.zip › Additional file 1 (.doc file).docx]

**Metabolic and transcriptional elucidation of the carotenoid biosynthesis pathway in peel and flesh tissue of loquat fruit during on-tree development**

Margarita Hadjipieri^1^, Egli C. Georgiadou^1^, Alicia Marin^2^, Huertas M. Diaz-Mula^2^, Vlasios Goulas^1^, Vasileios Fotopoulos^1^, Francisco A. Tomás-Barberán^2^, George A. Manganaris^1,*^

*^1^Cyprus University of Technology, Department of Agricultural Sciences, Biotechnology & Food Science, 3603 Lemesos, Cyprus*

*^2^Quality, Safety, and Bioactivity of Plant Foods, CEBAS-CSIC, P.O. Box 164, Espinardo, Murcia, Spain*

**Running title:** Carotenoid biosynthesis in loquat fruit

Corresponding author: G.A. Manganaris, Tel: (+357)25002307, Email: [george.manganaris@cut.ac.cy](mailto:george.manganaris@cut.ac.cy)

Additional file 1:

**Figure S1.**  HPLC chromatograms of saponified carotenoids in the peel and flesh of cv. ‘Obusa’ loquat fruits, at two maturity stages (S2 and S5) monitored at 450 nm. The peaks are numbered according to the elution sequence, as detailed in **Table 1**.

**Figure S2.** Relative expression levels of carotenoid biosynthesis genes (DXS, DXR, PSY1, PDS, ZDS, CRTISO, LCYB, CYCB, LCYE, BCH, ECH, ZEP and VDE) in loquat fruit (cv. ‘Obusa’) in peel during 5 developmental stages (S2-S6) (n = 3). Values that differ from the control (first developmental stage (S1) for peel tissue) with significance level P ≤ 0.05 are marked with *. Data are based on a statistical analysis of the means of three biological replications [22]. **Available in .xls file**

**Figure S3.** Relative expression levels of carotenoid biosynthesis genes (DXS, DXR, PSY1, PDS, ZDS, CRTISO, LCYB, CYCB, LCYE, BCH, ECH, ZEP and VDE) in loquat fruit (cv. ‘Obusa’) in flesh during 5 developmental stages (S2-S6) (n = 3). The expression of LCYE was undetectable from the S3 to S6 stages. Values that differ from the control (first developmental stage (S1) for flesh tissue) with significance level P ≤ 0.05 are marked with *. Data are based on a statistical analysis of the means of three biological replications [22]. **Available in .xls file**

**Table S1.** Previously published loquat carotenoids primers used for real-time RT-PCR analyses [10].

| \| **Gene** \| **Carotenoids**  **Primers** \| **Nucleotide sequence 5’-3’** \| **Product size (bp)** \| **Τa (°C)** \| **GenBank Accession Number** \| \| --- \| --- \| --- \| --- \| --- \| --- \| \| *Actin* \| *Actin-For* \| AATGGAACTGGAATGGTCAAGGC \| 227 \| 65 \| JN004223 \| \| *Actin-Rev* \| TGCCAGATCTTCTCCATGTCATCCCA \| \| *DXS* \| *DXS -For* \| GGTTCATCACTATTTGAAGA \| 206 \| 58 \| JN004208 \| \| *DXS -Rev* \| TCAAATTTGGCCACTCCATG \| \| *DXR* \| *DXR -For* \| CATCCAAACTGGAATATGGG \| 182 \| 65 \| JN004209 \| \| *DXR -Rev* \| GCCAGAACAGATGAATCCTG \| \| *PSY1* \| *PSY1-For* \| ACAGATGAGCTAGTGGATGG \| 186 \| 58 \| JN004210 \| \| *PSY1-Rev* \| CATTCCTTCTACCATGTCT \| \| *PDS* \| *PDS-For* \| AATGAGATGCTGACTTGGCC \| 263 \| 58 \| JN004211 \| \| *PDS-Rev* \| TTGGAACCGTGTTTCTCCTG \| \| *ZDS* \| *ZDS-For* \| AAGAAATGCAGTGGCTCTTGC \| 126 \| 65 \| JN004212 \| \| *ZDS-Rev* \| CCACCTTTGGACAAGAACCA \| \| *CRTISO* \| *CRTISO-For* \| AGCATTCCAACTGTTCTTGA \| 122 \| 58 \| JN004213 \| \| *CRTISO-Rev* \| TTCTTTGCCTCATAGTCCTT \| \| *LCYB* \| *LCYB-For* \| CAAACGGTGTTAAATTTCACCA \| 177 \| 65 \| JN004214 \| \| *LCYB-Rev* \| CCACTTGGTAACCTGGATTGTA \| \| *CYCB* \| *CYCB-For* \| AACCATGGATATCAGATTGCTC \| 134 \| 65 \| JN004215 \| \| *CYCB-Rev* \| GAATTACTAGTGCGCAAATAAGG \| \| *LCYE* \| *LCYE-For* \| ACTAGATTGTTCTTTGAGGA \| 148 \| 62 \| JN004216 \| \| *LCYE-Rev* \| CAACCGGAATCCAAGACCA \| \| *BCH* \| *BCH-For* \| GAGAAGGTCCGTTCGAGCT \| 172 \| 65 \| JN004217 \| \| *BCH-Rev* \| AGGCCATCATGGACAAACAT \| \| *ECH* \| *ECH-For* \| CGTTCTTCAAAGGTGTGGGA \| 155 \| 58 \| JN004218 \| \| *ECH-Rev* \| TCCAGCAGAGCAAACTGATC \| \| *ZEP* \| *ZEP-For* \| TACACTGGTATCGCAGATTT \| 128 \| 58 \| JN004219 \| \| *ZEP-Rev* \| TGAAACGCATACCACTGCAT \| \| *VDE* \| *VDE-For* \| TCTGATGTGGGAGAATTTCC \| 137 \| 62 \| JN004220 \| \| *VDE-Rev* \| TCATGCAATTGGCAATCAAA \| |
| --- | --- | --- | --- | --- | --- | --- | --- | --- | --- | --- | --- | --- | --- | --- | --- | --- | --- | --- | --- | --- | --- | --- | --- | --- | --- | --- | --- | --- | --- | --- | --- | --- | --- | --- | --- | --- | --- | --- | --- | --- | --- | --- | --- | --- | --- | --- | --- | --- | --- | --- | --- | --- | --- | --- | --- | --- | --- | --- | --- | --- | --- | --- | --- | --- | --- | --- | --- | --- | --- | --- | --- | --- | --- | --- | --- | --- | --- | --- | --- | --- | --- | --- | --- | --- | --- | --- | --- | --- | --- | --- | --- | --- | --- | --- | --- | --- | --- | --- | --- | --- | --- | --- | --- | --- | --- | --- | --- | --- | --- | --- | --- | --- | --- | --- | --- | --- | --- | --- |

**Table S2.** Weight (g), length (mm) and width (mm) (Mean ± standard error) of cv. ‘Obusa’ fruits during the on-tree developmental stages. The statistical analysis for each parameter (weight, length and width) shows the statistical difference between the 6 developmental stages.

| **Maturity stage** | **Weight (g)** | **Length (mm)** | **Width (mm)** |
| --- | --- | --- | --- |
| Immature green, S1 | 25.28±0.59 e | 48.83±0.69 d | 34.43±0.36 e |
| Mature green, S2 | 26.95±0.62 e | 51.11±0.53 c | 35.96±0.40 d |
| Breaker, S3 | 43.17±0.93 d | 57.03±0.45 a | 40.91±0.37 c |
| Half ripe, S4 | 50.94±1.23 c | 55.91±0.66 ab | 43.17±0.36 b |
| Fully ripe, S5 | 59.14±1.24 a | 56.85±0.51 a | 45.04±0.37 a |
| Over ripe, S6 | 55.28±1.05 b | 54.82±0.48 b | 44.05±0.36 ab |

| **Table S3.** Colour measurements for peel and flesh (Mean ± standard error) of cv. ‘Obusa’ fruits, over six developmental stages, expressed as L*a*b* values and a^*^/b^*^ ratio. The statistical analysis for each parameter (L*a*b* and a^*^/b^*^ ratio ) shows the statistical difference between the six developmental stages*.* |
| --- |
| \| **Maturity**  **stage** \| **Peel** \| \| \| \| **Flesh** \| \| \| \| \| --- \| --- \| --- \| --- \| --- \| --- \| --- \| --- \| --- \| \| **L*** \| **a*** \| **b*** \| **a*/** **b*** \| **L*** \| **a*** \| **b*** \| **a*/ b*** \| \| **Immature**  **Green, S1** \| 45.65±0.76e \| -16.73±0.22e \| 28.20±0.75e \| -0.60f \| 74.32±0.56a \| -11.51±0.33f \| 36.05±0.53a \| -0.32f \| \| **Mature**  **Green, S2** \| 52.58±0.69d \| -17.66±0.26f \| 36.06±0.77d \| -0.50e \| 66.71±0.58b \| -5.69±0.31e \| 30.87±0.63b \| -0.19e \| \| **Breaker, S3** \| 64.99±0.37a \| -9.81±0.38d \| 49.19±0.34a \| -0.20d \| 61.33±1.07c \| -1.84±0.19d \| 30.75±0.60b \| -0.06d \| \| **Half ripe, S4** \| 58.35±0.70c \| 3.22±0.29c \| 43.47±0.57b \| 0.08c \| 52.12±1.32d \| 3.95±0.26c \| 32.37±0.64b \| 0.12c \| \| **Fully ripe, S5** \| 58.78±0.62c \| 10.01±0.30b \| 41.52±0.53c \| 0.24b \| 53.62±0.77d \| 9.33±0.24b \| 34.94±0.48a \| 0.27b \| \| **Over ripe, S6** \| 60.61±0.61b \| 12.42±0.27a \| 43.58±0.58b \| 0.29a \| 53.46±0.69d \| 10.67±0.23a \| 36.67±0.52a \| 0.29a \| |

**Table S4.** Quantification of the identified carotenoids (**Table 1**) in peel and flesh for six maturity stages in loquat. Results are expressed as µg/100 g fresh weight (F.W.). Standard deviations (n=3) in parentheses.

| Peak nº | **Maturity stages (peel)** | | | | | | **Maturity stage (flesh)** | | | | | |
| --- | --- | --- | --- | --- | --- | --- | --- | --- | --- | --- | --- | --- |
|  | **S1** | **S2** | **S3** | **S4** | **S5** | **S6** | **S1** | **S2** | **S3** | **S4** | **S5** | **S6** |
| 1 | 44.3 (1.5) | 27.9 (3.3) | 24.4 (4.7) | 25.1 (2.4) | 26.9 (1.2) | 25.9 (2.9) | 13.8 (2.1) | 10.9 (0.4) | 10.0 (0.8) | 13.3 (0.2) | -- | -- |
| 2 | 26.1 (2.6) | 23.3 (1.3) | 16.4 (1.7) | 23.9 (2.1) | 22.9 (1.3) | 22.4 (0.2) | 13.6 (2.1) | 11.5 (0.1) | 10.5 (0.6) | 13.1 (0.2) | -- | -- |
| 3 | 15.6 (2.5) | 17.6 (0.3) | 16.4 (1.7) | 18.9 (1.2) | 26.8 (3.3) | 25.2 (4.6) | -- | -- | -- | 12.5 (0.2) | 18.1 (0.8) | 21.5 (1.6) |
| 5 | 17.3 (2.0) | 16.6  (0.9) | 15.4 (0.0) | 17.9 (0.6) | 21.4 (0.8) | 21.9 (1.4) | -- | -- | -- | -- | 16.3(0.4) | 19.2 (1.5) |
| 6 | -- | 16.0 (0.8) | 14.6 (0.8) | 17.5 (1.0) | 21.0 (1.1) | 21.3 (0.4) | -- | -- | -- | -- | -- | -- |
| 7 | -- | -- | 15.0 (0.8) | 17.3 (0.8) | 21.5 (1.8) | 21.2 (1.7) | -- | -- | -- | -- | -- | -- |
| 8 | 16.2 (2.45) | 15.9 (0.6) | 14.8 (0.2) | 17.7 (0.7) | 23.3 (2.0) | 23.2 (3.0) | -- | -- | -- | 12.3 (0.1) | 16.5 (0.9) | 19.1 (1.5) |
| 9 | 19.2 (1.9) | -- | 17.4 (0.8) | 19.9 (1.2) | 23.1 (1.6) | 22.8 (0.9) |  |  |  |  |  |  |
| 10 | 1621.5 (12.2) | 1228.6 (285.4) | 1241.5 (150.1) | 1068.2 (162.1) | 774.3 (70.4) | 688.4 (70.3) | 24.1 (6.0) | 11.5 (3.7) | -- | -- | -- | -- |
| 17 | 16.9 (2.7) | 19.5 (2. 9) | 20.2 (3.7) | 21.4 (1.3) | 24.6 (1.9) | 23.1 (0.2) | --- | -- | -- | -- | -- | -- |
| 18 | 19.4 (3.0) | 19.6 (2.4) | 21.0 (3.9) | 23.9 (2.0) | 23.7 (0.9) | 25.4 (0.7) | --- | -- | -- | -- | -- | -- |
| 19 | 26.3 (1.8) | 23.7 (0.6) | 20.5 (1.9) | 23.1 (2.9) | 26.1 (1.4) | 29.6 (2.8) | --- | -- | -- | -- | -- | -- |
| 24 | 85.3 (1.2) | 82.4 (5.7) | 72.3 (4.3) | 80.3 (3.9) | 98.8 (5.7) | 98.4 (2.3) | -- | -- | -- | 56.3 (0.3) | 82.1 (4.1) | 100.1 (6.2) |
| 25 | 68.6 (2.3) | 73.5 (7.0) | 67.8 (4.3) | 80.6 (4.2) | 101.9 (5.8) | 105.5 (1.9) | -- | -- | -- | 62.1 (0.3) | 84.4(5.6) | 95.7 (6.80) |
| 26 | 16.3 (2.2) | 16.1 (1.1) | 19.0 (6.2) | 27.2 (6.7) | 54.3 (5.0) | 58.4 (6.6) | -- | -- | -- | 65.4 (6.4) | 329.0 (13.3) | 595.0 (25.0) |
| 30 | -- | -- | 67.4 (4.4) | 81.6 (2.7) | 106.5 (6.5) | 109.9 (5.8) | -- | -- | -- | 56.0 (1.3) | 75.9 (3.0) | 87.8 (6.3) |
| 31 | 151.9 (30.9) | 188.7 (46.4) | 473.7 (42.2) | 502.8 (23.8) | 897.8 (54.5) | 1096.9 (38.7) | 80.9 (12.7) | 77.7 (4.0) | 124.6 (10.2) | 187.7 (30.9) | 253.2 (19.0) | 218.2 (48.2) |
| 32 | -- | -- | -- | -- | -- | -- | -- | -- | -- | -- | 17.6(1.2) | 21.4(1.8) |

**Table S5**. Relative expression levels of carotenoid biosynthesis genes (DXS, DXR, PSY1, PDS, ZDS, CRTISO, LCYB, CYCB, LCYE, BCH, ECH, ZEP and VDE) in loquat fruit (cv. ‘Obusa’) in peel during 5 developmental stages (S2-S6) (n = 3). Values that differ from the control (first developmental stage (S1) for peel tissue) with significance level P ≤ 0.05 are marked with bold letters. Data are based on a statistical analysis of the means of three biological replications [22]. **Available in .xls file**

**Table S6**. Relative expression levels of carotenoid biosynthesis genes (DXS, DXR, PSY1, PDS, ZDS, CRTISO, LCYB, CYCB, LCYE, BCH, ECH, ZEP and VDE) in loquat fruit (cv. ‘Obusa’) in flesh during 5 developmental stage (S2-S6) (n = 3). The expression of LCYE was undetectable from the S3 to S6 stages. Values that differ from the control (first developmental stage (S1) for flesh tissue) with significance level P ≤ 0.05 are marked with bold letters. Data are based on a statistical analysis of the means of three biological replications [22]. **Available in .xls file**
